# The first case report of an intraosseous epidermoid cyst in the distal phalanx of the index finger with infection resulting in single clubbing finger: A case report and review of the literature

**DOI:** 10.3389/fsurg.2022.1008358

**Published:** 2023-01-17

**Authors:** Ahmad Alhaskawi, Haiying Zhou, Yanzhao Dong, Jingtian Lai, Zewei Wang, Sohaib Hasan Abdullah Ezzi, Vishnu Goutham Kota, Mohamed Hasan Abdulla Hasan Abdulla, Zhenyu Sun, Hui Lu

**Affiliations:** ^1^ Department of Orthopedics, the First Affiliated Hospital, Zhejiang University, Hangzhou, China; ^2^ Department of Orthopedic, Zhejiang University School of Medicine, Hangzhou, China; ^3^ Department of Orthopedics of the Third Xiangya Hospital, Central South University, Changsha, China

**Keywords:** intraosseous epidermoid cyst, distal phalanx, clubbing, single clubbing finger, enchondroma

## Abstract

An intraosseous epidermoid cyst at the distal phalanx of the index finger is extremely rare. These cysts are asymptomatic unless ruptured, severely infected, or transformed into malignant squamous cell carcinoma. We present a case of a single clubbing finger in an adult diagnosed with an intraosseous epidermoid cyst in the distal phalanx of the left index finger with no history of pulmonary or cardiovascular diseases. Preoperative MRI showed an expansile lytic lesion with a sclerotic margin. Histopathological examination indicates that there is keratinous cell debris in the cyst with a wall of stratified squamous epithelium, which was the key to the correct diagnosis of an intraosseous epidermoid cyst. Written informed consent was obtained from the patient for publication of this case report and any accompanying images.

## Introduction

Intraosseous epidermoid cysts are also known as pseudotumors or keratin cysts. They were first reported in 1923 (1). They are rare benign bone lesions that occur mostly in the skull, face, neck, toe, and finger phalanges, especially the distal phalanges (2). It is quite challenging to diagnose this disease as it has no specificity in clinical and imaging presentations. An expansile lytic lesion with a sclerotic margin is usually shown in a radiological image (3). However, this radiological feature is shared with other diseases, such as enchondroma, giant cell tumors, subungual melanoma, aneurysmal bone cysts, and chondroma (4, 5). Therefore, a correct diagnosis is usually achieved by histopathologic examination (6, 7). A clubbing finger or Hippocratic finger is a finger deformity characterized by enlargement of the fingertips and the curved nails around the fingertips, which often signify poor cardiopulmonary function in patients (8). However, in this article, we report the first case of a single clubbing finger in an adult patient as a result of an infected intraosseous epidermoid cyst in the distal phalanx of the left index finger.

## Case report

**Figure 1:**
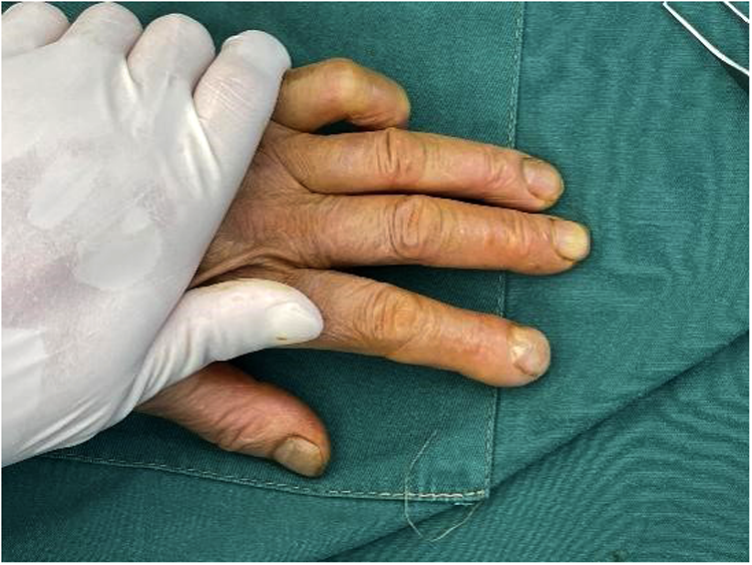
Preoperative view showing the swelling, redness, and tenderness on the eponychium of the left index finger.

In 2021, a 50-year-old female patient came to our outpatient department complaining of severe pain in the left index finger. Physical examination showed obvious swelling, redness, and tenderness on the eponychium of the left index finger, with nail root deformity, which looked similar to a clubbing finger, and the remaining fingers appeared normal (Figure 1). She denied a history of pulmonary and cardiovascular diseases except for a history of trauma in that she injured her left index finger 3 years ago and then the finger enlarged gradually and painlessly for the first few months after the injury. During this period, the fingernail of the left index finger fell off naturally twice. Six months ago, the patient visited a local hospital complaining of pain and intermittent infection, where the doctor considered the possibility of paronychia and gave her an antibiotic (celixin, the exact dosage, and usage are unknown) as the proper treatment. Unfortunately, the patient's pain was only relieved for a short period and became worse later on. Therefore, she visited our outpatient department for further treatment. After finishing the main outpatient check-up, a tumor at a distal phalanx of the left index finger was considered; thereby, the patient was hospitalized, and further examinations were required.

**Figure 2:**
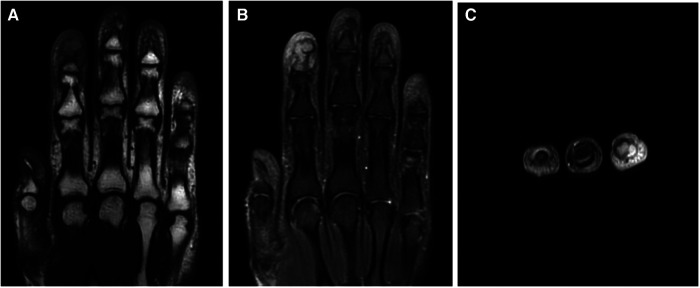
MRI of the distal phalanx of the left index finger. T1 (**A**) and T2 (**B,C**) showing a well-defined osteolytic lesion with a clear boundary.

**Figure 3:**
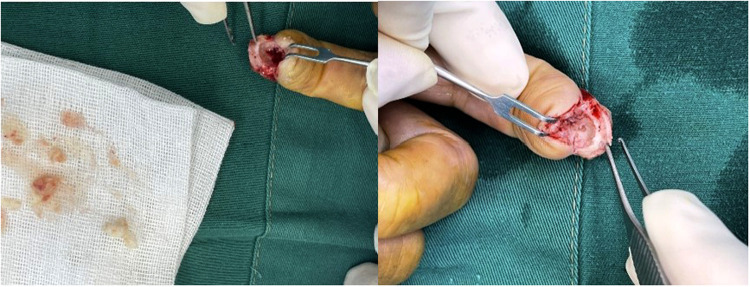
Creamy material removed from the cyst of the left index finger during the intraoperative curettage.

**Figure 4:**
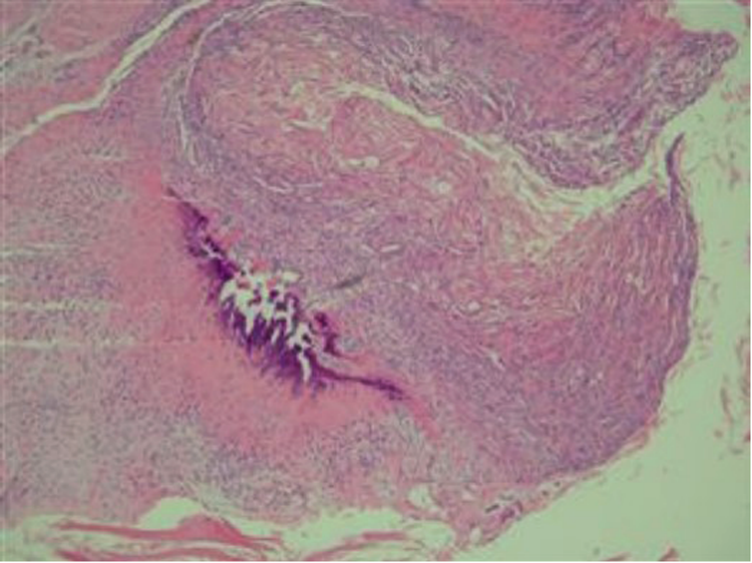
Histopathological examination revealing a wall of stratified squamous epithelium and keratinized material inside a cyst.

After admission, slightly limited mobility during flexion and circulation of the affected finger was observed and all blood tests were normal. Magnetic resonance imaging (MRI) revealed a lucent lytic lesion involving the distal phalanx that led to cortical thinning and bone expansion (Figure 2); thus, a tumor-like lesion was considered. Given her chronic severe pain and well-defined lesions on imaging, surgical excision was performed. After applying brachial plexus block anesthesia, we made a fish mouth flap incision in the distal phalanx of the left index finger, which allowed us to see the thinned phalangeal cortex and a cyst. A window was opened in the thinned cortex, where a creamy material was released, and then we performed curettage. After radical debridement, the skin was adjusted and the nail bed was sutured (Figure 3). Postoperative histopathological analysis of the lesion showed a cyst that contained a cavity of keratinized material and a wall of stratified squamous cells. These findings confirmed that the patient had an intraosseous epidermoid cyst (Figure 4), which previously had been misdiagnosed. Postoperative recovery was uneventful; the patient was allowed to have full motion of the left index finger 3 days after surgery. After 6 months of surgery, bone regeneration was noted radiographically and the patient was able to do full-weight exercises. After 1 year of clinical and radiological follow-up, the patient shows no sign of recurrence.

## Discussion

An intraosseous epidermal cyst or a squamous epithelial cyst is a lesion with keratinous debris surrounded by a wall of squamous epithelium in a bone. The cysts arise more frequently in men than women, and the left middle finger is involved most frequently (9). It is clinically presented as a painless, reddish, tender, and swollen lesion (2, 10, 11).

Many researchers have debated the real etiology of intraosseous epidermal cysts. The majority of previous studies found that trauma, such as crush injuries, is the common cause, and the epithelial cells could be embedded into the bone to form epidermoid cysts (11). Other studies mentioned congenital reasons why the inclusion of ectodermal tissue occurs during fetal development (12). All these etiologies ultimately point to the possibility of iatrogenic alteration of the epithelial cells within the bone, which leads to the formation of epidermoid cysts (11, 13).

Intraosseous epidermoid cysts are generally difficult to get diagnosed, depending on clinical examination and radiological findings, because their appearances and other characteristics are similar to those of other diseases, such as enchondroma, giant cell tumors, synovial cysts, aneurysmal bone cysts, and gout (14). Radiological imaging of an intraosseous epidermoid cyst shows a well-defined lytic lesion with circumscribed margin in a bone (2, 4). This imaging evidence lacks the specificity required for the definitive diagnosis of an intraosseous epidermoid cyst. Therefore, a histopathological test of the lesion tissue is the final resort to confirm the diagnosis of intraosseous epidermoid cysts among the various differential diagnoses. The histopathological analysis presents that an intraosseous epidermoid cyst contains keratinous cell debris surrounded by a wall of the stratified squamous epithelium (2). The most reliable treatments for intraosseous epidermoid cysts are curettage, which avoids recurrence, and bone graft, which reconstructs the wound (3, 15).

The noteworthy point found in our patient is that only her affected finger, a single finger, showed the clubbing finger changes. Clubbing finger changes often appear in multiple fingers and are a sign of multiple severe diseases, such as lung cancer, cystic fibrosis, sarcoidosis, cirrhosis, and cardiovascular diseases, which occur gradually over the years (16–19). Single clubbing finger occurs extremely rarely. A few cases have been reported, and all of them were linked to enchondroma, a type of benign bone tumor (19–21), except one formed as a result of superficial acral fibromyxoma (22) (Table 1).

**Table 1:** Cases with single clubbing fingers.

Gender	Age	Injured site	Case	Treatment	Follow-up period	References
Female	18	Left middle finger	Single clubbing finger caused by enchondroma	N/A	N/A	(21)
Female	23	Left ring finger	Single clubbing finger caused by enchondroma	Curettage and then the cavity filled with synthetic bone substitute	No evidence of recurrence, with a nearly normal-looking finger	(20)
Female	50	Right thumb	Single clubbing finger caused by enchondroma	Curettage and then the cavity filled with synthetic bone substitute	Performed full motion of the thumb, no recurrence after surgery	(19)
Female	58	Right index finger	Single clubbing finger caused by superficial acral fibromyxoma	Complete excision of the lesion	N/A	(22)

The mechanism of the clubbing finger remains ambiguous. A few theories, such as vascular endothelial growth factor (VEGF) (23), platelet-derived growth factor (PDGF) (24), and neurocirculatory reflex (25), are postulated to be participating in the formation of clubbing finger. Clubbing does not occur suddenly, and it has different stages. It starts as periungual erythema with a soft sensation, and then the angle between the proximal nail fold and the nail bed will increase. Clubbing causes the nail to be convex in shape, the skin at the fingertip to be shiny, and the possibility of hyperextension of the interphalangeal joint (26).

## Conclusion

We here present the first case of a single clubbing finger associated with intraosseous epidermoid cysts. This case report intends to inspire clinicians that the differential diagnosis of the clubbing finger, especially in the case of a single clubbing finger, should include intraosseous epidermoid cysts.

## Data Availability

The original contributions presented in the study are included in the article/Supplementary Material; further inquiries can be directed to the corresponding author/s.

## References

[1] Hammann VI. Traumatische epithelcysten an den fingerknochen. Deutsche Ztschrift Für Chirurgie. (1930) 223(4–5):308–17.

[2] Simon K Leithner A Bodo K Windhager R. Intraosseous epidermoid cysts of the hand skeleton: a series of eight patients. J Hand Surg Eur. (2011) 36(5):376–8. 10.1177/1753193411401987 21372056

[3] Belusa M. Intra-osseous epidermoid cyst. Handchir Mikrochir Plast Chir. (1991) 23(4):200–1. 1937184

[4] Kozlowski K Azouz EM Campbell J Marton D Morris L Padovani J Sprague P Beluffi G Berzero GF Cherubino P. Primary bone tumours of the hand. Report of 21 cases. Pediatr Radiol. (1988) 18(2):140–8. 10.1007/BF02387558 3353148

[5] Patel K Bhuiya T Chen S Kenan S Kahn L. Epidermal inclusion cyst of phalanx: a case report and review of the literature. Skeletal Radiol. (2006) 35(11):861–3. 10.1007/s00256-005-0058-0 16416148

[6] Carroll RE. Epidermoid (epithelial) cyst of the hand skeleton. Am J Surg. (1953) 85(3):327–34. 10.1016/0002-9610(53)90617-5 13030935

[7] Musharrafieh RS Tawil AN Saghieh SS Macari G Atiyeh BS. Epidermoid cyst of the thumb. Orthopedics. (2002) 25(8):862–3. 10.3928/0147-7447-20020801-20 12195917

[8] Spicknall KE Zirwas MJ English JC 3rd. Clubbing: an update on diagnosis, differential diagnosis, pathophysiology, and clinical relevance. J Am Acad Dermatol. (2005) 52(6):1020–8. 10.1016/j.jaad.2005.01.006 15928621

[9] Nakajo M Ohkubo K Nandate T Nagano Y Shirahama H Nakajo M. Intraosseous epidermal cyst of the distal phalanx of the thumb: radiographic and magnetic resonance imaging findings. Radiat Med. (2005) 23(2):128–32. PMID: 15827532

[10] Fisher ER Gruhn J Skerrett P. Epidermal cyst in bone. Cancer. (1958) 11(3):643–8. 10.1002/1097-0142(195805/06)11:3<643::AID-CNCR2820110328>3.0.CO;2-O 13523575

[11] Schajowicz F Aiello CL Slullitel I. Cystic and pseudocystic lesions of the terminal phalanx with special reference to epidermoid cysts. Clin Orthop Relat Res. (1970) 68:84–92. 10.1097/00003086-197001000-00014 4313226

[12] Niccoli C Mambelli V. Intra-osseous epithelial cyst of the phalanx. Arch Putti Chir Organi Mov. (1978) 29:395–404. 757494

[13] Hamad AT Kumar A Anand Kumar C. Intraosseous epidermoid cyst of the finger phalanx: a case report. J Orthop Surg. (2010) 2010:340–2. 10.1177/230949900601400322 17200542

[14] Ruchelsman DE Laino DK Chhor KS Steiner GC Kenan S. Digital intraosseous epidermoid inclusion cyst of the distal phalanx. J Hand Microsurg. (2010) 2(1):24–7. 10.1007/s12593-010-0001-z 23129949 PMC3452980

[15] Kurosawa K Kobayashi R Takagishi K. Distal phalangeal reconstruction for recurrent intraosseous epidermoid cyst of the finger—a case report. Hand Surg. (2011) 16(3):375–7. 10.1142/S0218810411005734 22072479

[16] Burcovschii S Aboeed A. Nail clubbing, in StatPearls. Treasure island (FL): StatPearls Publishing LLC (2022). 30969535

[17] Yanardag H Tetikkurt C Bilir M. Finger clubbing and cirrhosis in a sarcoidosis patient. Monaldi Arch Chest Dis. (2019) 89(3):1–4. 10.4081/monaldi.2019.1141 31631644

[18] Epstein O Dick R Sherlock S. Prospective study of periostitis and finger clubbing in primary biliary cirrhosis and other forms of chronic liver disease. Gut. (1981) 22(3):203–6. 10.1136/gut.22.3.203 7227854 PMC1419499

[19] Hui Lu Qiang Chen Hui Shen Hu Yang Weifeng Ji. Enchondroma in the distal phalanx of the thumb with infection resulting in clubbing felon. Hangzhou China: International Journal of Clinical and Experimental Medicine (IJCEM) (2018)

[20] Nishio J Naito M. Single clubbed finger caused by an enchondroma of the distal phalanx: an unusual clinical presentation. Hand Surg. (2012) 17(3):405–8. 10.1142/S0218810412720422 23061956

[21] Coto-Segura P Mallo-Garcia S Braña A Santos-Juanes J. Single hippocratic (clubbed) finger revealing an underlying enchondroma. J Am Acad Dermatol. (2008) 59(2 Suppl 1):S34–5. 10.1016/j.jaad.2007.07.041 18625376

[22] Crestani L Fasciani IA Kakizaki P Valente NYS. Case for diagnosis. Single-digit clubbing. An Bras Dermatol. (2020) 95(4):524–6. 10.1016/j.abd.2020.01.006 32448714 PMC7335881

[23] Atkinson S Fox SB. Vascular endothelial growth factor (VEGF) – a and platelet-derived growth factor (PDGF) play a central role in the pathogenesis of digital clubbing. J Pathol. (2004) 203(2):721–8. 10.1002/path.1565 15141388

[24] Dickinson CJ Martin JF. Megakaryocytes and platelet clumps as the cause of finger clubbing. Lancet. (1987) 2(8573):1434–5. 10.1016/S0140-6736(87)91132-9 2891996

[25] Gold AH Bromberg BE Herbstritt JG Stein H. Digital clubbing: a unique case and a new hypothesis. J Hand Surg Am. (1979) 4(1):60–6. 10.1016/S0363-5023(79)80106-9 759505

[26] Altman RD Tenenbaum J. Hypertrophic osteoarthropathy. Textbook of Rheumatology (2009). p. 1606–8.

